# Vascular Endothelial Growth Factor expression in peripheral blood of patients with pregnancy induced hypertension syndrome and its clinical significance

**DOI:** 10.12669/pjms.303.4558

**Published:** 2014

**Authors:** Yanfang Ren, Huiling Wang, Haixia Qin, Jun Yang, Yuhong Wang, Shan Jiang, Ying Pan

**Affiliations:** 1 Yanfang Ren, Department of Obstetrics & Gynecology, The First Affiliated Hospital of Xinxiang Medical University, Weihui 453100, China.; 2 Huiling Wang, Department of Obstetrics & Gynecology, The First Affiliated Hospital of Xinxiang Medical University, Weihui 453100, China.; 3 Haixia Qin, Department of Obstetrics & Gynecology, The First Affiliated Hospital of Xinxiang Medical University, Weihui 453100, China.; 4 Jun Yang, Department of Obstetrics & Gynecology, The First Affiliated Hospital of Xinxiang Medical University, Weihui 453100, China.; 5 Yuhong Wang, Department of Obstetrics & Gynecology, The First Affiliated Hospital of Xinxiang Medical University, Weihui 453100, China.; 6 Shan Jiang, Department of Obstetrics & Gynecology, The First Affiliated Hospital of Xinxiang Medical University, Weihui 453100, China.; 7 Ying Pan, The Third First Affiliated Hospital of Xinxiang Medical University, Xinxiang 453003, China.

**Keywords:** Cardiovascular, Hypertension, Pregnancy complications, Vascular endothelial growth factor

## Abstract

***Objective:*** This study was conducted was to detect vascular endothelial growth factor (VEGF) levels in peripheral blood of patients with pregnancy-induced hypertension (PIH) syndrome and to investigate VEGF correlation with PIH occurrence.

***Methods:*** Double-antibody enzyme-linked immunosorbent assay and fluorescent quantitative polymerase chain reaction were used to detect VEGF levels in the peripheral blood of non-pregnant women (normal group, 30 cases), normal pregnant women (pregnancy group, 30 cases) and PIH patients (PIH group, 30 cases).

***Results:*** VEGF level in the pregnancy group was significantly higher than in the normal group, and the difference between these two groups was significant (*P* < 0.001). In the pregnancy group, VEGF reached the maximum level at the metaphase stage of pregnancy and started to decrease at advanced pregnancy. VEGF level in the PIH group was significantly lower than in the pregnancy group at advanced pregnancy (*P* < 0.01), and VEGF level significantly and gradually decreased with PIH aggravation (*P *< 0.05).

***Conclusions:*** The significant decrease of VEGF level after pregnancy was possibly an important factor of PIH pathogenesis.

## INTRODUCTION

Pregnancy induced hypertension syndrome (PIH) is a high-incidence disease of pregnant women that can damage the heart, brain, kidney and other organs. PIH reduces uterine and placental blood perfusion and can severely impair foetal survival. The possibility of risk occurrence increases in a PIH patient suffering from congestive heart failure.^1^^–^^3^ VEGF, a cytokine on the surface of vascular endothelial cells, macrophages and nurse cells, is involved in vascular formation and reconstruction; VEGF combines with a specific receptor to increase vasopermeability.^4^^–^^6^ VEGF is significant in the maintenance of vascular integrality and normal permeability and has a critical regulatory function in pathological physiology associated with hyperplasia of vascular endothelial cells.

VEGF is mainly expressed on the surface of placental syncytiotrophoblast cells and invasive chorionic trophoblast cells during pregnancy; VEGF is particularly expressed at the vascular bud site at early pregnancy, during which syncytiotrophoblast cells are abundant.^7^^–^^10^ VEGF has an important function in vascular endothelial damage, and various growth factors have an important function in disease. However, information on PIH expression is lacking. In this study, we used fluorescent quantitative polymerase chain reaction (PCR) and enzyme-linked immunosorbent assay (ELISA) to detect VEGF level in the peripheral blood of patients with PIH and investigated the participation of VEGF in PIH incidence.

## METHODS

***Objects and grouping:*** 1) The PIH group comprised 30 PIH patients hospitalised in the Gynaecology and Obstetrics Department of our hospital from May 2010 to April 2011. The diagnosis code and classification criteria used were the same as those in a previous study.^11^ Among these 30 PIH cases, 14, 10 and 6 cases had mild, moderate and severe PIH, respectively. The average age was 26.2±5.6 years old, and the average pregnancy duration was 35.3±3.1 weeks. 2) The pregnancy group comprised 30 randomly selected cases of normal healthy pregnant women admitted in our hospital at the same period. Ten cases of pregnant women in the early, metaphase and advanced pregnancy stages were included. The average age was 25.3±4.9 years old, and the average pregnancy duration was 27.6±4.7 weeks. 3) The normal group comprised 30 randomly selected cases of normal non-pregnant women examined in the hospital at the same period, and average age was 25.2±3.8 years old. Women in all three groups did not have histories of hypertension, heart disease, diabetes mellitus and liver and kidney diseases. This study was conducted in accordance with the declaration of Helsinki and with approval from the Ethics Committee of the First Affiliated Hospital of Xinxiang Medical University. All participants provided written informed consent.

***Specimen acquisition*** ***and*** ***processing:*** Elbow vein blood (2 ml) was drawn from all subjects on an empty stomach and the blood was injected into non-anticoagulation glass tubes. Venous blood was centrifuged for ten minutes at 4,000 r/min and 4 °C to obtain the serum. The serum was stored at –70°C.

***ELISA detection:*** ELISA was performed according to the instructions on the VEGF ELISA kit (Roch, USA).

***Fluorescent quantitative*** ***reverse transcription-polymerase chain reaction (*** ***RT*** ***–*** ***PCR*** ***):*** The total RNA of nucleated cells in peripheral blood was extracted, and absorbance was detected with an ultraviolet spectrophotometer at 260 and 280 nm to calculate optical density (OD)260/OD280. The ratio value should be between 1.75 and 1.95. For fluorescent quantitative RT–PCR test, the relative quantitative method was used to detect changes in gene expression. The △△cycle threshold (CT) method was used for quantitative analysis if the feasibility test verified that the difference of amplification oblique line between target gene and internal reference was less than 0.1. In addition, real-time RT–PCR reaction was performed according to the manufacturer’s instructions.

***Reaction conditions:*** A total of 40 cycles were performed at 95 °C for 10 s, 95 °C for 5 s and 60 °C for 34 s. VEGF PCR reaction and another PCR reaction with GAPDH as the internal reference were simultaneously conducted. The relative expression amount of VEGF-mRNA in the specimen was calculated according to the following formulas: △CT (target gene) = target gene CT - internal control gene CT and △△CT = △CT (target gene) - △CT (standard value). The relative total amount of target gene was 2^-^^△△^^CT^. The CT value represented the number of cycles necessary for the fluorescence signal in each reaction tube to reach the set threshold. In addition, the CT value was negatively related to VEGF mRNA expression level; an increase in CT value indicated a corresponding decrease in VEGF mRNA level. The VEGF and internal primer sequences used are shown in Table-I.

***Statistical Analysis:*** All data were expressed as $\bar{X}$±s. Two-sample t test was used for comparison between groups. Statistical software SPSS 15.0 was used. Significant difference was set at *P* < 0.05.

## RESULTS

***ELISA result:*** As shown in Table-II, the pregnancy group showed significantly higher serum VEGF level than the non-pregnant normal group, and the difference between these two groups was extremely significant (*P* < 0.001). In addition, VEGF level of pregnant women was highest at metaphase stage and lowest at advanced pregnancy stage. The average VEGF level of the PIH group was significantly lower than that of the pregnancy group. The VEGF level of the PIH group was significantly lower (*P* < 0.01) than that of the pregnancy group at advanced pregnancy. The VEGF level gradually decreased with PIH aggravation. The VEGF level in severe PIH was significantly lower than in moderate PIH, and the difference was statistically significant (*P *< 0.05).

***Fluorescent quantitative RT*** ***–*** ***PCR:*** The relative quantitative method 2^-^^△△^^CT ^was used to compare VEGF mRNA expression level in peripheral blood of women in normal non-pregnant, PIH and pregnancy groups. The VEGF mRNA expression level of normal non-pregnant women was considered as the standard. The relative expression levels of VEGF mRNA in peripheral blood of the pregnancy and PIH groups were compared. Statistical analysis showed significant differences in peripheral blood VEGF mRNA expression levels among the three groups (*P *< 0.05). The PIH and pregnancy groups showed significantly higher VEGF mRNA expression levels in peripheral blood than the non-pregnant normal group (*P* < 0.05). PIH group showed significantly lower VEGF mRNA expression level than the pregnancy group at advanced pregnancy (*P* < 0.01). VEGF level gradually decreased with PIH aggravation, and VEGF level at severe PIH was significantly lower than at moderate PIH (*P* < 0.05) (Table-III). This finding was in agreement with the ELISA results.

**Table-I:** Primer sequences used in this study

*Primer*	*Sequence (5′-3′)*	*Product length(bp)*
VEGF	AAGATCCGCAGACGTGTAAATGTT CGGCTTGTCACATGCAAGTA	100
GAPDH	CTTAGCACCCCTGGCCAAG GATGTTCTGGAGAGCCCCG	150

**Table-II:** Comparison of VEGF level among the PIH, Pregnancy and control group ($\bar{X}$±s).

*Group*	*Case(n)*	*VEGF(ng/L)*
Normal	30	12.98±3.99
pregnancy	30	149.39±27.15*
Early	10	145.04±23.69
Mid-term	10	183.84±49.02
Late	10	118.37±34.29
PIH	30	62.25±24.33^＃^
Mild	10	87.26±11.24
Moderate	10	57.64±11.74
Severe	10	35.44±9.37^△^

*P* < 0.001 verse normal group;

＃ *P *< 0.01 verse late Pregnancy in the Pregnancy group; *P *< 0.05

△ moderate PIH group.

**Table-III:** VEGF mRNA expression among the PIH, pregnancy and control group

*Group*	*Case(n)*	*△* *CT value*	*△△* *CT*	*2* ^-^^△△^^CT^	*P value*
Normal	30	12.72±1.47	0.00±1.47	1.00	
Pregnancy	30	8.97±0.95	-3.75±0.95	13.45(6.96~25.99)	*P *< 0.05
PIH	30	10.50±1.03	-2.22±1.03	4.66(2.28~9.51)	*P *< 0.05

## DISCUSSION

VEGF, also known as vascular permeability factor (VPF), is a functional glycoprotein with high biological activity. VEGF undergoes specific combination with receptors FIT-1 and KDR and subsequently promotes vascular endothelial cell division and proliferation, increases vasopermeability, regulates the apoptosis signal transduction pathway of endothelial cells and increases cell resistance to the apoptosis process.^12^^,^^13^ Studies showed that VEGF has an important function in the implantation process of fertilised ovum and in vascular formation in the placental growth process during the entire pregnancy period. VEGF mRNA expression level in placental tissue and VEGF level in peripheral blood obviously increased after pregnancy.^14^^,^^15^ In the present study, VEGF level in serum of normal pregnant women was significantly higher than in non-pregnant women, and the maximum level was observed at metaphase pregnancy. Serum VEGF level started to decrease at advanced pregnancy stage.

The possible mechanisms underlying the increase in VEGF level during pregnancy include the following:^16^^–^^18^ 1) trophoblastic villi were relatively hypoxic during the development process at early pregnancy; 2) maintenance of adequate blood circulation was required during placental growth; and 3) blood and oxygen supply was required in the foetal growth process. Previous studies confirmed that the expressions of VEGF and its receptors in placental tissue were detected during the entire pregnancy period and that expression intensity increased with progressing pregnancy^19^. These findings suggest that VEGF has an important function in placental vascular development and function maintenance. Increasing VEGF level helps maintain the integrality and normal permeability of maternal blood vessels and is significant for regulating maternal cardiovascular adaptation to pregnancy. In addition, VEGF has a critical regulatory function in the pathological physiology associated with hyperplasia of vascular endothelial cells.^19^

VEGF level in peripheral blood was higher in PIH patients than in non-pregnant women, but was significantly lower than in homochronous normal pregnant women. VEGF is mainly expressed in placental syncytiotrophoblast and invasive chorionic trophoblast cells during pregnancy, especially at the vascular bud site at early pregnancy, during which syncytiotrophoblast cells were strongly positive.^20^ The decrease in VEGF level in the peripheral blood of PIH patients suggests that VEGF affects the differentiation and proliferation of nurse cells, causes invasive functional disorder of nurse cells and affects the physiological changes of spiral arterioles. In addition, VEGF level in the serum of PIH patients is related to disease condition. VEGF levels decrease with aggravating disease condition, suggesting that the decrease in VEGF level down regulates placental local VEGF expression in PIH patients and directly inhibits local vascular growth and development to induce the occurrence of PIH.

Therefore, a decrease in VEGF synthesis and release may be an important factor in PIH pathogenesis. The resulting vascular endothelial damage possibly worsens with lower VEGF levels; therefore, PIH disease condition worsens. VEGF level in the serum of PIH patients is negatively related to disease condition. The decrease in VEGF level becomes more significant with increasing PIH severity, suggesting that the decrease in VEGF synthesis and release may be an important factor in PIH pathogenesis.

This experiment is a preliminary study. A large sample size is recommended for future studies. VEGF expression and the reference range of serum VEGF should also be investigated in the future.
